# PrsA2 (CD630_35000) of *Clostridioides difficile* Is an Active Parvulin-Type PPIase and a Virulence Modulator

**DOI:** 10.3389/fmicb.2018.02913

**Published:** 2018-12-04

**Authors:** Can Murat Ünal, Mareike Berges, Nathiana Smit, Cordelia Schiene-Fischer, Christina Priebe, Till Strowig, Dieter Jahn, Michael Steinert

**Affiliations:** ^1^Institut für Mikrobiologie, Technische Universität Braunschweig, Braunschweig, Germany; ^2^Türk-Alman Üniversitesi, Moleküler Biyoteknoloji Bölümü, Istanbul, Turkey; ^3^Helmholtz-Zentrum für Infektionsforschung, Braunschweig, Germany; ^4^Institut für Biochemie und Biotechnologie, Martin-Luther-Universität Halle-Wittenberg, Halle, Germany; ^5^Braunschweig Integrated Centre of Systems Biology, Braunschweig, Germany

**Keywords:** *Clostridioides difficile*, PrsA2, peptidyl-prolyl-*cis/trans*-isomerase, resistance, germination, colonization

## Abstract

*Clostridioides difficile* is the main cause for nosocomial antibiotic associated diarrhea and has become a major burden for the health care systems of industrial countries. Its main virulence factors, the small GTPase glycosylating toxins TcdA and TcdB, are extensively studied. In contrast, the contribution of other factors to development and progression of *C. difficile* infection (CDI) are only insufficiently understood. Many bacterial peptidyl-prolyl-*cis/trans-*isomerases (PPIases) have been described in the context of virulence. Among them are the parvulin-type PrsA-like PPIases of Gram-positive bacteria. On this basis, we identified CD630_35000 as the PrsA2 homolog in *C. difficile* and conducted its enzymatic and phenotypic characterization in order to assess its involvement during *C. difficile* infection. For this purpose, wild type CdPrsA2 and mutant variants carrying amino acid exchanges mainly in the PPIase domain were recombinantly produced. Recombinant CdPrsA2 showed PPIase activity toward the substrate peptide Ala-Xaa-Pro-Phe with a preference for positively charged amino acids preceding the proline residue. Mutation of conserved residues in its active site pocket impaired the enzymatic activity. A PrsA2 deficient mutant was generated in the *C. difficile* 630*Δerm* background using the ClosTron technology. Inactivation of *prsA2* resulted in a reduced germination rate in response to taurocholic acid, and in a slight increase in resistance to the secondary bile acids LCA and DCA. Interestingly, in the absence of PrsA2 colonization of mice by *C. difficile* 630 was significantly reduced. We concluded that CdPrsA2 is an active PPIase that acts as a virulence modulator by influencing crucial processes like sporulation, germination and bile acid resistance resulting in attenuated mice colonization.

## Introduction

The Gram-positive obligate anaerobe *Clostridioides difficile* was first isolated in 1935 from neonates and identified as part of their natural intestinal microbiota. In 1978 it was recognized as the causative agent of enterocolitis and pseudomembraneous colitis following the introduction of clindamycin, a broad-band lincosamin antibiotic against Gram-negative anaerobes (Bartlett et al., 1977; Lusk et al., 1978). In industrialized countries, *C. difficile* infection (CDI) has become a major burden in health care facilities in the last two decades with increasing incidence numbers in community-associated cases (Rupnik et al., 2009; Hopkins and Wilson, 2018). The most common severe form of CDI is pseudomembranous colitis which is a strong inflammation that goes along with fever, massive tissue destruction of the large intestine and leucocytosis (Barbut et al., 2007; Rupnik et al., 2009).

*Clostridioides difficile* infection is mainly mediated by two enterotoxins (TcdA and TcdB) that are essential for developing the disease (Burke and Lamont, 2014). These toxins are taken up by endocytosis, translocate into the host cell cytosol and exert their activity by glycosylating and thereby inactivating small GTPases in human enterocytes. This leads to the collapse of the actin cytoskeleton dynamics resulting in the distortion of the gut epithelial barrier and inflammation (Rupnik et al., 2009; Chandrasekaran and Lacy, 2017). Apart from its enterotoxins, several other virulence factors that contribute to disease severity and host colonization have been described for *C. difficile* and analyzed to different extents. These include, among others, the binary toxin CDT (*C. difficile* toxin) that is present in 5–6% of historic human isolates, extracellular proteases, surface layer proteins, several adhesins like a fibronectin binding protein (Fbp68) or a collagen binding protein (CbpA), flagella and type IV pili (Geric et al., 2004; Janoir, 2016; Ünal and Steinert, 2016; Péchiné et al., 2018). Furthermore, the bile acid status of the infected host was shown to clearly determine the course and outcome of CDI as patients display a clear shift from antibacterial to less antibacterial bile acids due to the disturbances of their indigenous microbiome (Theriot et al., 2016). Besides this, the arsenal of virulence factors and mechanisms that contribute to host colonization, disease outbreak and dissemination is still largely unexplored.

Peptidyl-prolyl-*cis/trans*-isomerases (PPIases) are ubiquitous proteins that can be divided into three major classes: the FK-506 binding proteins (FKBPs), cyclophilins and parvulins (Schiene-Fischer et al., 2013; Dunyak and Gestwicki, 2016). Despite their amino acid sequence and structural differences all classes have in common that they catalyze the rate limiting isomerization of peptidyl-prolyl bonds during protein folding (Fischer et al., 1984; Fischer and Bang, 1985; Siekierka et al., 1989; Rahfeld et al., 1994). By this, they contribute to protein stability, activity and translocation in bacteria (Behrens-Kneip, 2010; Lyu and Zhao, 2015). Physiologically, PPIases participate in stress tolerance, protein homeostasis or secretion. In many instances PPIases have been shown to contribute to virulence in Gram-negative and Gram-positive bacteria (Ünal and Steinert, 2014). The most prominent examples are the macrophage infectivity potentiator (Mip) of *Legionella pneumophila*, and PrsA2 of *Listeria monocytogenes* (Cahoon and Freitag, 2014; Rasch et al., 2014).

PrsA2 of *L. monocytogenes* (LmPrsA2) is a parvulin-type PPIase that is a lipoprotein and localizes to the cytoplasmic membrane. Like its homolog PrsA from *Bacillus subtilis*, it facilitates folding and efficient secretion of extracellular proteins (Kontinen et al., 1991; Vitikainen et al., 2004; Cahoon and Freitag, 2014). LmPrsA2 was first identified in a transposon mutant study as a factor promoting the virulence related trait haemolysis (Zemansky et al., 2009). Subsequently, it was shown to promote the activity or efficient secretion of several other virulence factors like the metalloprotease Mlp, the phospholipase PC-LPC, listeriolysin O or the actin-binding and nucleating protein ActA. Thus, it was influencing the outcome of infection in cellular and animal models (Alonzo and Freitag, 2010; Alonzo et al., 2011; Forster et al., 2011). Furthermore, LmPrsA2 homologs have been described in other major Gram-positive pathogens like *Staphylococcus aureus, Streptococcus equi* or *S. suis* in the context of virulence (Ikolo et al., 2015; Cincarova et al., 2016; Jiang et al., 2016; Wiemels et al., 2017). Accordingly, in this study we analyzed the closest homolog of LmPrsA2 in *C. difficile*, CD630_35000, in respect to its biochemistry as well as contribution to physiology and infection.

## Materials and Methods

### Bacterial Strains and Culture

Bacterial strains and plasmids that were used in this study are listed in Table 1. *C. difficile* 630*Δerm* and its derivatives were cultured in BHIS medium (brain-heart infusion broth (Carl Roth GmbH) supplemented with 5 g/L yeast extract (BD Bacto^TM^) and 1 g/L L-cysteine (Sigma-Aldrich) under anaerobic conditions (95% N_2_/5% H_2_). If necessary, 5 μg/mL erythromycin or 15 μg/mL thiamphenicol were added. *Escherichia coli* and *Bacillus megaterium* were cultured in LB medium supplemented with 100 μg/mL ampicillin, 10 μg/mL tetracycline or 30 μg/ml chloramphenicol. All antibiotics were purchased from Sigma-Aldrich. Media were solidified by adding 1.5% (w/v) agar when needed.

**Table 1:** Bacterial strains and plasmids used in this study.

Name	Features	Reference
*C. difficile* str. 630*Δerm*	Erythromycin-sensitive derivative of strain 630	DSMZ^†^ (DSM 28645), (Hussain et al., 2005)
*C. difficile* str. 630*ΔermΔprsA2*	*cd35000::ClosTron*	This study
*E. coli* DH10β	Strain for cloning and plasmid propagation, F^-^ *mcrA* *Δ*(*mrr*-*hsd*RMS-*mcr*BC) Φ80d*lac*Z*Δ*M15 *Δlac*X74 *end*A1 *rec*A1 *deo*R *Δ*(*ara, leu*)7697 *ara*D139 *gal*U *gal*K *nup*G *rps*L λ^-^	Grant et al., 1990
*B. megaterium* MS941	Strain for recombinant production, deficient of the major secreted protease (*ΔnrpM*)	Wittchen and Meinhardt, 1995
pSP_YocH_-hp	Vector for recombinant production in *B. megaterium*, P_xylA_-(-35^+^ rbs^+^)-*sp*_yocH_-*mcs*-his6, Tc^R^, Amp^R^	Stammen et al., 2010


*^†^German Collection of Microorganisms and Cell Cultures.*

### Recombinant Production of CdPrsA2 and Its Single Amino Acid Substitution Mutants

The *prsA2* gene without the first 66 bp encoding for the N-terminal signal peptide was amplified with the primers 3500_For and 3500_Rev. The PCR product was cloned into the production vector pSP_YocH_-hp using the restriction sites *Eag*I and *Spe*I yielding a C-terminally His-tagged protein that is N-terminally coupled to the signal peptide of YocH of *B. subtilis* (Stammen et al., 2010). Conserved amino acids, which were suspected to be involved in PPIase activity were substituted by alanine. This was done by inverse PCR utilizing the original production construct as template and primers with the respective codon changes. The linearized plasmids were gel purified, phosphorylated with T4 polynucleotide kinase (NEB) and re-ligated with T4 ligase (NEB) according to manufacturer’s instructions. Plasmids carrying the desired mutations were selected in *E. coli* DH10β and verified by sequencing. After propagation and validation in *E. coli* DH10β, the protoplasts of the strain *B. megaterium* MS941 were transformed using purified plasmid DNA as described previously (Biedendieck et al., 2011). For recombinant production, an overnight culture of the production strain was prepared, and the next morning refreshed 1:100 in 300 ml LB medium containing 10 μg/mL tetracycline. The cells were induced at an OD_600nm_ of ∼0.4 by the addition of sterile filtered D-xylose (Carl Roth GmbH) at a final concentration of 0.5 % (w/v). After 6 h the cells were removed (3000 *g*, 15 min, RT), and the supernatant was sterile filtered as pSP_YocH_-hp facilitates the secretion of the recombinantly produced protein into the culture supernatant. His-tagged PrsA2 and its variants were purified from the supernatant using Protino^®^ Ni-TED columns (Macherey-Nagel) following the manufacturer’s instructions. Protein yield and purity were analyzed by SDS-page. Correct secondary structure folding of recombinant proteins was confirmed by circular dichroism. Primers used in this study are listed in Table 2.

**Table 2:** Primers used in this study.

Name	Sequence^†^	Features	Reference
PrsA2(wt)_For	TATactagtAGTAAAGGAGAAACTGTGGC	*Spe*I	This study
PrsA2(wt)_Rev	TATcggccgAGATCCACGAGGTACTAAGATTGTGATTTTATTTAT	*Eag*I, Thrombin site	This study
PrsA2(K188A)_For	**GCA**ACAGTAGATGATAATAACAAGCC		This study
PrsA2(K188A)_Rev	CAATAAAATATGAGAAGCTTCTAC		This study
PrsA2(D232A)_For	ATATTCACAA**G**CTACTTCAGCAAG		This study
PrsA2(D232A)_Rev	TTTTTTGCTACTTTTGCAAAATCCTC		This study
PrsA2(L241A)_For	**GCA**GGATTCTTTTCAAGGGGTCAA		This study
PrsA2(L241A)_Rev	TTTACCACCATCACTTGCTGA		This study
PrsA2(M249A)_For	GGTCAA**GC**GGTTGCTGAATTTG		This study
PrsA2(M249A)_Rev	CCTTGAAAAGAATCCTAATTTACC		This study
PrsA2(F253A)_For	**GCA**GAAGATGCTGCTTTTTCTATGAA		This study
PrsA2(F253A)_Rev	TTCAGCAACCATTTGACCCC		This study
PrsA2(T271A)_For	**GCT**CAATATGGATACCACATAATTAAAG		This study
PrsA2(T271A)_Rev	TTCAACTAAATCAGATACTTCACC		This study
PrsA2(Y273A)_For	GAAACTCAA**GC**TGGATACCACAT		This study
PrsA2(Y273A)_Rev	AACTAAATCAGATACTTCACCCTTT		This study
PrsA2(H276A)_For	TGGATAC**GCT**ATAATTAAAGTGACAG		This study
PrsA2(H276A)_Rev	TATTGAGTTTCAACTAAATCAGATAC		This study
EBS universal primer	CGAAATTAGAAACTTGCGTTCAGTAAAC		Heap et al., 2007
ErmRAM_F	ACGCGTTATATTGATAAAAATAATAGTGGG		Heap et al., 2007
ErmRAM_F	ACGCGTGCGACTCATAGAATTATTTCCTCCCG		Heap et al., 2007


*^†^Highlighted are the bases for the respective alanine exchange, restriction sites are kept in lowercase.*

### Protease-Free PPIase Assay

Protease-free PPIase assays were performed as previously described (Zoldák et al., 2009). Briefly, measurements of the PPIase activity were done in 35 mM HEPES pH 7.8 at 10°C in a HP8452 UV/Vis spectrophotometer using the substrate peptide Suc-Ala-Ala-Pro-Phe-pNA. The time courses were followed at 330 nm after jumping from the peptide stock solution in 0.55 M LiCl/TFE into the final buffer solution. Data analysis was performed by single exponential non-linear regression. Measurements were performed at different concentrations of PrsA2 in triplicate. Determination of k_cat_/K_m_ was performed by evaluation of the linear dependency of k_enz_ from the concentration of PrsA2. Relative errors of the measurements were calculated using the standard errors of the linear regression. Substrate specificity was determined using substrate peptides of the form Abz-Ala-Xaa-Pro-Phe-pNA with Xaa representing different proteinogenic amino acids. Measurements were done in a Fluoromax 4 fluorescence spectrophotometer after jumping from the peptide stock solution in 0.55 M LiCl/TFE into the final buffer solution. The time courses were followed at 416 nm after excitation at 316 nm. Relative errors were calculated using the standard errors of the measurements in triplicate. Data were analyzed by ANOVA.

### Construction of CD630_35000 (*prsA2*) Destruction Clones Using ClosTron

A synthetic vector containing the region of *prsA2* (CD630_35000) was designed with the help of the ClosTron website^1^ using the Perutka algorithm (Perutka et al., 2004). *E*. *coli* CA434 was transformed with customized vectors for mating with *C. difficile* 630*Δerm* cells as described previously (Purdy et al., 2002; Heap et al., 2009, 2010). Mutants were selected on BHIS containing 5 μg/mL erythromycin and confirmed by PCR and sequencing using gene specific primers.

### Determination of Toxin Concentration in Supernatants

For determining the toxin production, exponentially growing cultures were adjusted to an OD_600 nm_ of 0.05 in 10 mL of fresh BHIS and grown at 37°C under anaerobic conditions. After 48 h, the culture supernatants of 2 mL of each culture were harvested (5000 *g*, 5 min, RT) and sterile filtered using 0.2 μm syringe filters. Toxin concentrations in culture supernatants were determined using the *Clostridium difficile* Toxin A OR B ELISA Kit of tgcBIOMICS (tgc-E002-1) following the manual instructions.

### Preparation of Spores

Spores of *C. difficile* were prepared with slight modifications as previously described (Sorg and Sonenshein, 2008). Bacteria were grown over night in BHIS medium, and 100 μL were spread on BHIS-agar plates, which were incubated for 10 days at 37°C under anaerobic conditions. Bacteria and spores were collected using a sterile loop and suspended in 2 mL ice cold H_2_O_dd_. Spores were separated from vegetative cells by layering this suspension onto 10 ml of sterile 50% (w/v) sucrose solution and centrifugation (3200 *g*, 4°C, 25 min). This step was repeated until a spore purity of > 95% was reached as assessed microscopically. Spores were washed five times with H_2_O_dd_ (5000 *g*, 4°C, 10 min), resuspended in 1 mL of H_2_O_dd_, and stored at 4°C.

### Assessment of Sporulation Rates

The spore formation was assessed microscopically as described previously (Edwards and McBride, 2016). Briefly, 100 μL of an exponentially growing culture were spread out on sporulation agar plates [63 g Bacto peptone (BD Difco), 3.5 g proteose peptone (BD Difco), 0.7 g (NH_4_)_2_SO_4_, 1.06 g Tris base, 11.1 g BHI extract (Roth) and 1.5 g yeast extract (BD Difco) in 1000 mL H_2_O_dd_, autoclave and add 3 ml 10% (w/v) of sterile filtered L-cysteine (Sigma-Aldrich)]. The plates were incubated at 37°C under anaerobic conditions. After 24 h, a loop full of bacteria were resuspended and fixed for 15 min at room temperature in 100 μL of PBS containing 2% (w/v) paraformaldehyde. Two to five μL of these suspensions were dropped onto a microscope slide, pictures of at least three different fields were taken, and the number of vegetative cells and spores were determined.

### Germination Assays

Spore germination in response to the bile acids taurocholate (TCA) or deoxycholate (DCA) was measured optically as described previously (Sorg and Sonenshein, 2008, 2009). Briefly, spores were heat activated at 60°C for 30 min, adjusted to an OD_600__nm_ of 0.3–0.5 in 100 μL BHIS containing 5 mM TCA or 1 mM DCA in a 96-well plate, and immediately transferred to a VarioSkan^TM^ (ThermoFisher) plate reader at room temperature. Germination of the spores resulted in a decrease in the optical density, which was recorded every 2 min for 20 min.

### Resistance Assays

Overnight cultures were refreshed 1:100 in 10 mL BHIS medium, cultured to an OD_600__nm_ of 0.8–1.0, and adjusted to 0.1 in BHIS medium. Bile acids (20 mM stock solution in DMSO) or antibiotics (5 mM stock solution in DMSO) were serially diluted 1:1 in a 96-well plate in 100 μL BHIS containing 10% (v/v) DMSO. Following this, 100 μL of the bacterial solution were added into each well resulting in a final OD of 0.05 and a final DMSO concentration of 5% (v/v). Bacteria were grown for 12–16 h at 37°C under anaerobic conditions, and the final OD was measured at 600 nm using a VarioSkan^TM^ (ThermoFisher) plate reader. Medium containing 5% (v/v) DMSO served as background.

### Mouse Colonization Studies

Mice for *C. difficile* infection were bred at the Helmholtz Centre for Infection Research (Braunschweig, Germany) under enhanced specific pathogen free conditions. CHOW AND IVC. 16–20 weeks old mice (GENDER) were treated with an intraperitoneal injection of clindamycin (10mg/kg body weight, Sigma) and 24 h later orally infected with 1000 spores of the indicated strains of *C. difficile*. Twenty four hours after infection, fecal samples were collected and mice were euthanized to obtain cecum and colon content. Samples were weighed and homogenized, after which aliquots of homogenized contents were heated for 20 min at 65°C to kill vegetative cells. To determine CFU of vegetative cells and spores, non-heated and heated samples were plated on CLO agar plates (BioMerieux, 43431), which were for the heated samples supplemented with taurocholate (0.1% w/v, Roth) to induce germination of spores. Plates were incubated for 3 days at 37°C in an anaerobic environment (Anaerocult A, Merck). All experiments were performed in strict accordance with the German Recommendation of the Society for Laboratory Animal Science (GV-SOLAS) and the European Health Recommendations of the Federation of Laboratory Animal Science Associations. The animal protocol was approved by the “Niedersächsisches Landesamt für Verbraucherschutz und Lebensmittelsicherheit”: (33.9-42502-04-14/1415).

## Results

### *C. difficile* Encodes Proteins With Strong Amino Acid Sequence Similarity to PPIases

**FIGURE 1:**
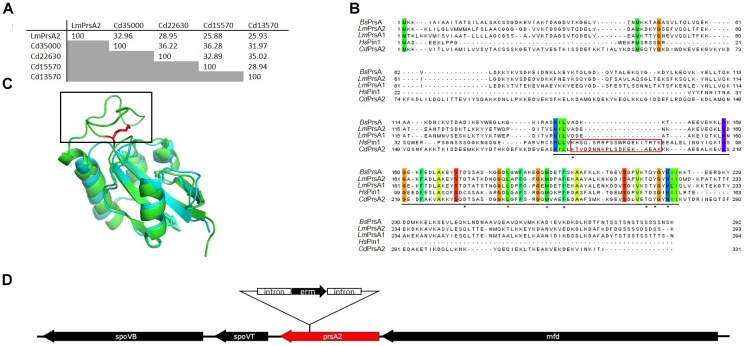
CD630_35000 is the closest homolog of PrsA2 of *L. monocytogenes* in *C. difficile* with unique features. **(A)** Similarity analysis of all putative parvulins of *C. difficile* with the virulence associated parvulin LmPrsA2 of *L. monocytogenes* shows that CD630_35000 (CdPrsA2) displays the highest similarity to LmPrsA2. **(B)** Alignment of amino acid sequences of CdPrsA2 (Q180Z8), LmPrsA1 (Q71ZM6) and LmPrsA2 (Q71XE6) of *L. monocytogenes*, BsPrsA (P24327) of *B. subtilis* and HsPin1 (Q13526) of humans reveals highly conserved amino acids, especially in the PPIase domain (underlined). The alignment was performed using the T-Coffee webserver and visualized by Jalview 2.10.3b1. Amino acids that are conserved to 100% are highlighted by color coding according to Taylor (Taylor, 1986; Notredame et al., 2000; Waterhouse et al., 2009). Amino acids that have been mutated in CdPrsA2 for enzymatic studies are marked by an asterisk. A variable loop that aligns with the human HsPin1 and is absent in all other bacterial PrsA proteins is highlighted in red. **(C)** Structural alignment of HsPin1 (pdb:1NMV) and BsPrsA (pdb: 4WO7) reveals a loop (highlighted in red) in the human parvulin that has no homology in BsPrsA but aligns with CdPrsA2 (see also **B**). Shown in red is the side chain of the catalytically important amino acids K63 in HsPin1 that corresponds to K188 in CdPrsA2. **(D)** Shown is the genetic localization of the 996 base pairs long ORF of CD630_35000 (CdPrsA2) in the genome of *C. difficile* strain 630*Δerm* with the integration site of the *ClosTron* construct between bases 262 and 263. Shown are also the downstream adjacent genes *spoVB* and *spoVT* as well as the upstream adjacent gene *mfd*.

We screened the reference genome of *C. difficile* str. 630 (NC_009089.1) for the presence of genes encoding parvulin homologs and identified four putative candidates (Table 3). Of those four, two contained putative secretion signals (SP) that can be recognized by the signal peptidase II as predicted by LipoP 1.0 (Juncker et al., 2003; Rahman et al., 2008). This is similar to *L. monocytogenes* where, in contrast to the model organism *B. subtilis*, two membrane localized PrsA-homologs are present (Cahoon et al., 2016). In order to determine the closest homolog of the virulence associated PrsA2 of *L. monocytogenes* (LmPrsA2), we performed an amino acid sequence alignment with Clustal Omega (Sievers et al., 2011). For this, we used the sequences of LmPrsA2 and the four putative parvulin-type PPIases from *C. difficile*, CD630_13570, CD630_15570, CD630_22630 as well as CD630_35000. Out of those, CD630_35000 had with 32.96% identity the highest similarity on amino acid level to LmPrsA2 and separated in this respect clearly from the remaining parvulins of *C. difficile* (Figure 1A). Further amino acid sequence comparisons of CdPrsA2 (CD630_35000) with LmPrsA1, LmPrsA2, BsPrsA and the human parvulin Pin1 (HsPin1) identified highly conserved amino acids, mostly in the well-defined PPIase domain (Figure 1B). This amino acid sequence alignment shows the close relatedness between BsPrsA, LmPrsA1 and LmPrsA2. Interestingly, CdPrsA2 contained a stretch of 21 amino acids (K188-K208) that was absent in the other bacterial parvulins. Interestingly, HsPin1 contains a stretch of amino acids which is approximately of the same length. Overlaying an available structural model of the PPIase domain of BsPrsA with HsPin1 showed that this stretch corresponded to a flexible loop in HsPin1 that is absent in BsPrsA (Figure 1C). The 996 bp long CdPrsA2 gene is localized on the chromosome between the gene for RNA polymerase removal factor Mfd upstream and the gene encoding the highly conserved forespore-specific transcription factor SpoVT downstream (Figure 1D) (Asen et al., 2009; Willing et al., 2015).

**Table 3:** Putative parvulin-type PPIases of *C. difficile.*

	Locus tag^‡^				
	
Accession number^†^	630	630*Δerm*	R20291	SP^§^
Q18BM5	13570	01513	1199	–
Q18C77	15570	01726	1406	+
Q185D5	22630	02497	2162	–
Q180Z8	35000	03813	3337	+


*^†^Uniprot IDs, ^‡^in the reference genomes NC_009089.1 (str. 630), CP016318.1. (str. 630Δerm) and FN545816.1 (str. R20291), ^§^signal peptide as determined by SignalP 4.1.*

### CdPrsA2 Is an Active Parvulin

**FIGURE 2:**
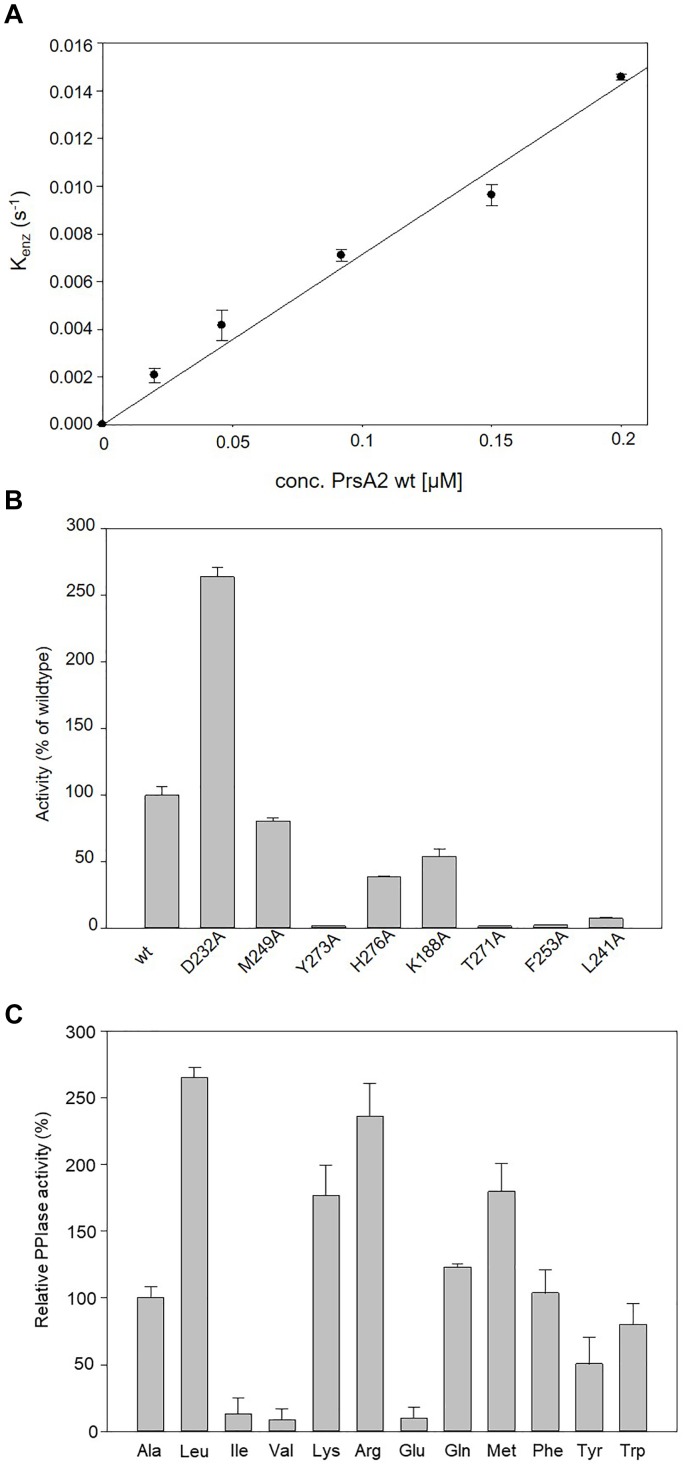
PPIase activity profile of CdPrsA2. **(A)** Determination of the catalytic efficiency of wtPrsA2 (k_cat_/K_m_ 6.78 × 10^4^ M^-1^ s^-1^) by evaluation of the linear dependency of k_enz_ from the concentration of the protein. The *cis/trans* isomerization of the PPIase substrate Suc-Ala-Ala-Pro-Phe-pNA was measured by the protease-free PPIase activity assay. The time courses were followed using a HP 8452 diode array spectrophotometer at 330 nm after jumping from the peptide stock solution in LiCl/TFE into the final buffer solution at 10°C. Measurements were done in 35 mM HEPES pH 7.8. Each data point represents the mean of three independent measurements. **(B)** Substitution of selected conserved amino acids in the PPIase domain by alanine revealed that especially the amino acids in the catalytic core Y273, T271, F253 and L241 are involved in the PPIase activity. Substitution of Y273 and T271 reduced the catalytic efficiency to 1.5% of the wild type protein, whereas the alanine substitutions of F253 and L241 yielded catalytic efficiencies of 2.3 and 7.4%, respectively. Moderate changes were observed when M249 or H276 were replaced by alanine, which decreased catalytic efficiencies to 80.5 and 38.5%, respectively. Replacement of the non-canonical K188 by alanine yielded a variant with only 53.8% residual efficiency. Substituting D232 by alanine resulted in 264% increased PPIase activity compared to wild type protein. **(C)** Relative PPIase activities of CdPrsA2 for substrates with different amino acids N-terminal to proline compared to its activity towards Abz-Ala-Ala-Pro-Phe-pNA. Determination of k_cat_/K_m_ was performed by evaluation of the linear dependency of k_enz_ from the concentration of CdPrsA2. Catalytic efficiencies were increased to 264, 236, or 176% for substrates with leucine or the positively charged amino acids arginine or lysine preceding proline, respectively. In contrast, the catalytic efficiency dropped to 9.9% or to 8.4%, when substrate peptides containing glutamate or valine were used, respectively. Data are means and SEM of three independent measurements. Statistical significance was determined by one-way analysis of variance (ANOVA), (^∗^*p* ≤ 0.05).

For evaluating whether CdPrsA2 is an active parvulin, we recombinantly produced a C-terminally His-tagged CdPrsA2 lacking its putative signal peptide and lipid modification as well as its single alanine-exchange mutant variants in *B. megaterium* MS941 and performed *in vitro* protease-free PPIase assays using the substrate peptide Abz-Ala-Ala-Pro-Phe-pNA. Wild type CdPrsA2 was active in the protease-free PPIase assay with a catalytic efficiency (k_cat_/K_m_) of 6.78 ± 0.41 × 10^4^ M^-1^s^-1^ (Figure 2A and Table 4). In order to functionally identify amino acid residues involved in the observed enzymatic activity, conserved residues of the PPIase domain were deduced from the outlined amino acid sequence comparisons (D232, L241, M249, F253, T271, Y273, H276). Additionally, K188 was selected due to its possible functional homology to K63 in HsPin1 (Zhou et al., 2000) (Figure 1B). Among the eight amino acid replacements, Y273A, T271A, and F253A were the most deleterious ones with catalytic efficiencies dropping to 1.06 ± 0.24 × 10^3^ M^-1^s^-1^, 1.06 ± 0.10 × 10^3^ M^-1^s^-1^, 1.61 ± 0.23 × 10^3^ M^-1^s^-1^, respectively, and resulting in an activity loss of about 98% compared to the wild type protein. This was followed L241A with 92% reduced activity and a catalytic efficiency of 5.02 ± 0.50 × 10^3^ M^-1^s^-1^ (Figure 2B). Two substitution mutants, namely M249A and H276A, revealed activity losses of about 20 and 70%, respectively. Also, exchanging the non-canonical K188 by alanine caused a loss in activity by about 45% to a k_cat_/K_m_ value of 3.65 02 ± 0.40 × 10^4^ M^-1^s^-1^. In contrast, substituting D232 by an alanine caused an opposite effect and increased the catalytic efficiency of the enzyme 2.6-fold to 1.80 ± 0.05 × 10^5^ M^-1^s^-1^ (Figure 2B).

**Table 4:** Relative catalytic activity of alanine substitution mutants of CdPrsA2.

PrsA2	k_cat_/K_m_ (M^-1^ s^-1^)
Wild type	(6.78 ± 0.41) × 10^4^
D232A	(1.80 ± 0.05) × 10^5^
M249A	(5.49 ± 0.16) × 10^4^
Y273A	(1.06 ± 0.24) × 10^3^
H276A	(2.61 ± 0.06) × 10^4^
K188A	(3.65 ± 0.40) × 10^4^
T271A	(1.06 ± 0.10) × 10^3^
F253A	(1.61 ± 0.23) × 10^3^
L241A	(5.02 ± 0.50) × 10^3^


Next, we analyzed the substrate preference of CdPrsA2 by using the wild type protein with variants of the substrate peptide Abz-Ala-Xaa-Pro-Phe-pNA containing different amino acid residues Xaa preceding the proline residue (Table 5). The catalytic efficiency of CdPrsA2 for a substrate peptide with leucine instead of alanine was with 8.5 × 10^5^ M^-1^s^-1^ about 2.6-fold increased. We concluded a clear preference of CdPrsA2 toward positively charged amino acids preceding proline, as the peptides containing arginine or lysine were isomerized 2.3-fold and 1.76-fold faster resulting in k_cat_/K_m_ values of 7.58 × 10^5^ M^-1^s^-1^ and 5.67 × 10^5^ M^-1^s^-1^, respectively. In agreement with this, the catalytic efficiency for a substrate peptide with the negatively charged glutamate was reduced by around 90% to 3.2 × 10^4^ M^-1^s^-1^. Peptides containing isoleucine or valine were less suitable substrates as their k_cat_/K_m_ values were reduced by 87 and 92% to 4.26 × 10^4^ M^-1^s^-1^ and 2.7 × 10^4^ M^-1^s^-1^, respectively (Figure 2C).

**Table 5:** Substrate specificity of wild type CdPrsA2 protein.

Xaa^a^	k_cat_/K_m_ (M^-1^ s^-1^)
Ala	(3.21 ± 0.27) × 10^5^
Leu	(8.50 ± 0.67) × 10^5^
Ile	(4.26 ± 0.51) × 10^4^
Val	(2.70 ± 0.23) × 10^4^
Lys	(5.67 ± 1.30) × 10^5^
Arg	(7.58 ± 1.85) × 10^5^
Glu	(3.20 ± 0.26) × 10^4^
Gln	(3.96 ± 0.08) × 10^5^
Met	(5.77 ± 1.22) × 10^5^
Phe	(3.33 ± 0.58) × 10^5^
Tyr	(1.63 ± 0.32) × 10^5^
Trp	(2.58 ± 0.40) × 10^5^


*^*a*^Amino acids used to replace the position preceding proline in the substrate peptide Abz-Ala-Xaa-Pro-Phe-pNA.*

### CdPrsA2 Is Involved in Stress Responses

**FIGURE 3:**
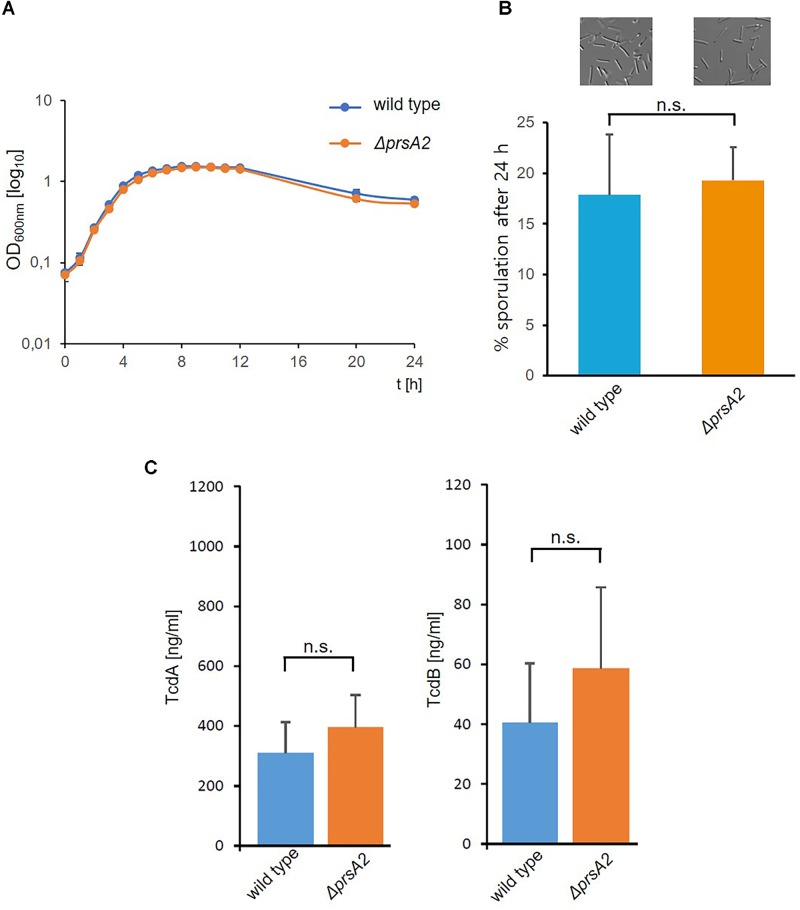
Deletion of CdPrsA2 does not affect growth, sporulation or toxin production. **(A)** Growth in BHIS medium was measured over 24 h every 90 min for the first 14 h. Shown is the mean and standard deviation of two separate experiments performed in duplicates. **(B)** Sporulation of 630*Δerm* (wild type) and its isogenic *ΔprsA2::ClosTron* mutant (*ΔprsA2*) was induced on sporulation plates for 24 h, and monitored microscopically. The number of spores was put in relation to the vegetative cells in order to assess the sporulation rate in percentage. Shown are the results of three independent experiments. **(C)** Toxin concentrations in 48 h old culture supernatants were measured by ELISA and revealed no substantial differences between 630*Δerm* (wild type) and its isogenic *ΔprsA2::ClosTron* mutant (*ΔprsA2*).

**FIGURE 4:**
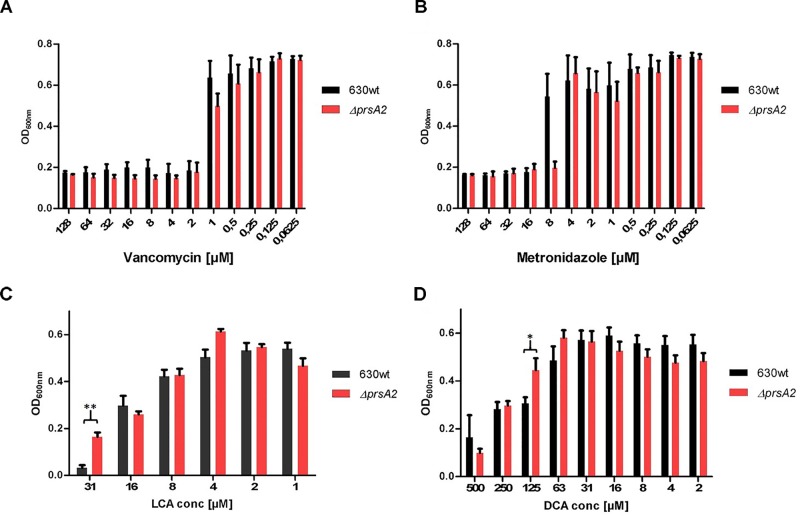
CdPrsA2 confers metronidazole resistance and renders the bacteria sensitive to secondary bile acids. **(A)** Vancomycin is equally effective against vegetative *C. difficile* 630*Δerm* (wild type) and its isogenic *ΔprsA2::ClosTron* mutant with a minimal inhibitory concentration (MIC) of ≥ 1 μM. **(B)** In case of metronidazole the *ΔprsA2::ClosTron* mutant showed increased sensitivity as the MIC dropped from ≥ 8 μM to ≥4 μM. In the absence of PrsA2 *C. difficile* can tolerate higher concentrations of the secondary acids **(C)** litocholic acid (LCA) and **(D)** deoxycholic acid (DCA) as the MICs for the substances increase from ≥ 16 μM to ≥31 μM, and from ≥63 μM to ≥125 μM, respectively. Shown are the mean and standard deviation of three independent experiments performed in duplicates. Significance was calculated using unpaired two-sided Student’s *t-*test (^∗^*p* ≤ 0.05, ^∗∗^*p* ≤ 0.01).

Next, we analyzed its physiological role by disrupting the CdPrsA2 gene using the ClosTron technology. The ClosTron element was found inserted between bps 262 and 263 (Figure 1D). The mutant revealed no obvious growth defect in rich BHIS-medium (Figure 3A). Similarly, the spore forming capacity and the toxin titres of stationary liquid cultures were comparable between the *prsA2::ClosTron* mutant and its isogenic wild type (Figures 3B,C). PPIases are typically involved in various stress responses in bacteria. Consequently, we analyzed the contribution of CdPrsA2 to different stress responses of *C. difficile*. As stressors we used the two most common therapeutic antibiotics, vancomycin and metronidazole, and the two main secondary bile acids of the healthy intestine, lithocholic acid (LCA) and deoxycholic acid (DCA) that inhibit vegetative cell growth. When treated with vancomycin, no significant difference between the wild type strain and its isogenic *prsA2::ClosTron* mutant was detected (Figure 4A). In contrast, a significant reduction in resistance from 16 to 8 μM metronidazole was observed for the mutant strain (Figure 4B). The mutant strain was found less susceptible toward bile acids, with 31 μM of LCA (Figure 4C) and 125 μM of DCA (Figure 4D), concentrations at which the vegetative growth of the wild type strain was significantly impaired.

### Response to Germination Inducing Bile Salts Is Impaired in Spores Lacking CdPrsA2

**FIGURE 5:**
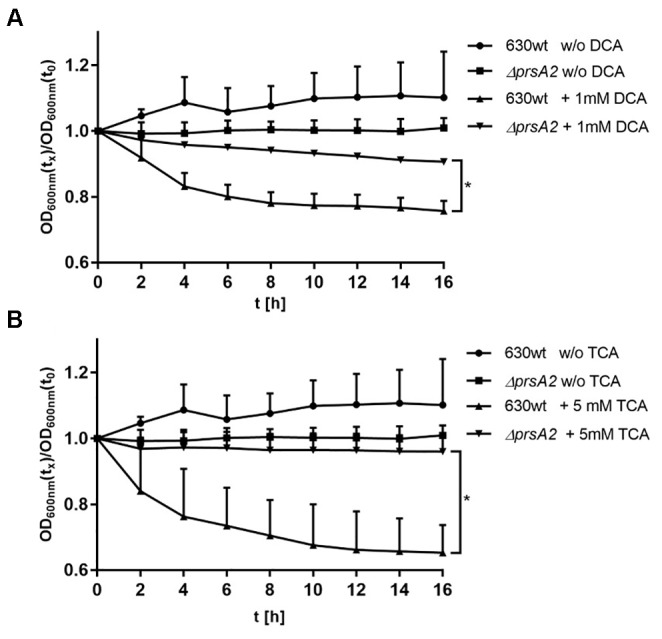
CdPrsA2 influences germination efficiency of *C. difficile* spores. Germination of *C. difficile* 630*Δerm* (wild type) and its isogenic *ΔprsA2::ClosTron* mutant (*ΔprsA2*) in the presence of **(A)** 1 mM deoxycholic acid (DCA) or **(B)** 5 mM taurocholic acid (TCA) was assessed in 96-wells by measuring the change in optical density of the spore suspension at OD_600__nm_ over 20 min. Plotted is the change in optical density relative to the starting OD at t_0_. Shown are the means and SEM of three measurements performed with spores of two different preparations. Statistical significance was calculated using Student’s *t*-test (^∗^*p* ≤ 0.05).

The germination of spores is the crucial step for colonization and developing acute CDI. Accordingly, we evaluated the response of spores derived from the wild type strain and isogenic *prsA2::ClosTron* mutant. For this purpose, we used the bile acid DCA at a concentration of 1 mM and sodium taurocholate (TCA) with 5 mM and monitored the initial stages of germination by measuring the drop in OD at 600 nm every 2 min for 20 min after the addition of the respective bile acid. The change in relative OD indicated a significant response of the wild type strain to both bile acids. The relative OD decreased by about 20% during treatment with 1 mM DCA (Figure 5A) and by about 25% with 5 mM TCA (Figure 5B) until it reached a plateau at 20 min. In contrast, spores of the *prsA2* deficient mutant did not respond to both bile acids in the same time frame and showed less than 5% loss in OD (Figures 5A,B).

### CdPrsA2 Contributes to Colonization of Mice

**FIGURE 6:**
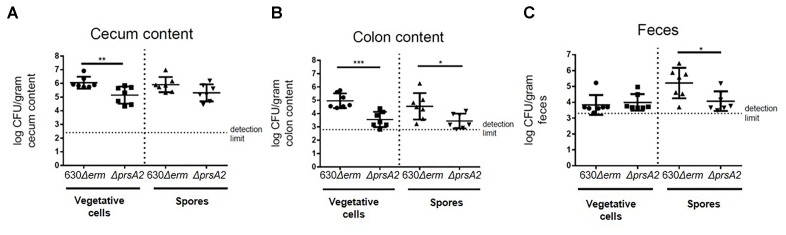
CdPrsA2 is important for colonization of mice. Twenty to twenty four weeks old mice old clindamycin treated mice were infected with 1000 spores of either *C. difficile* 630*Δerm* and its isogenic *ΔprsA2::ClosTron* mutant (*ΔprsA2*) per mouse, sacrificed after 24 h, and **(A)** cecum, **(B)** colon contents as well as **(C)** feces were collected. The number of vegetative cells and spores in each compartment were determined by plating out serial dilutions on CLO plates (bioMérieux) with or without 0.1% (w/v) TCA. Samples were heated prior to plating out for the determination of spore numbers. Significantly higher cfu of vegetative cells could be isolated from cecum and colon of mice infected with the wild type as compared with the *ΔprsA2* mutant. Furthermore, significantly less spores of the mutant were recovered in the colon and feces samples. Significance was calculated using unpaired two-sided Student’s *t-*test (^∗^*p* ≤ 0.05, ^∗∗^*p* ≤ 0.01, ^∗∗∗^*p* ≤ 0.001).

Having seen the differential effects in *C. difficile* regarding bile acid responses, we evaluated the *prsA2* deficient mutant in a mouse colonization model. For this purpose, 4 months old mice that had been treated with clindamycin were infected orally with 1000 spores of *C. difficile* str. 630*Δerm* or its isogenic *prsA2::ClosTron* mutant. The mice were sacrificed 24 h after infection, and the cecum and colon contents as well as the feces were collected. The colonization of the mice was assessed by determining the colony forming units (cfu) of each strain on selective agar. Mice infected with wild type spores yielded a higher bacterial burden in their cecum and colon compared to the mutant strain (Figures 6A,B). In the cecum, 10^6^ cfu/g of vegetative cells were recovered. This was about 10-fold and by this significantly higher (*p* ≤ 0.01) than in mice infected with the PrsA2-deficient mutant (Figure 6A). This difference was even higher in the colon, where 10^5^ cfu/g of vegetative cells of wild type compared to about 5 × 10^3^ cfu/g (*p* ≤ 0.001) of vegetative cells of the mutant were recovered (Figure 6B). In contrast, no difference in the count of vegetative cells was observed between the strains in the feces, where about 3.5 × 10^3^ cfu/g were recovered for both strains (Figure 6C). The spore numbers of the wild type were with 4.5 × 10^4^ vs. 3.2 × 10^3^ cfu/g in the colon and 5.2 × 10^5^ vs. 10^4^ cfu/g in feces significantly higher for the wild type (Figures 6B,C). No significant differences in the spore counts were measured in the cecum (Figure 6A).

## Discussion

The multi-resistant pathogen *C. difficile* is a steadily growing problem for the health care systems of industrialized countries as its spread goes along with the broad use of antibiotics in communities with a demographic change toward a care-intensive population. While the acute disease is mainly caused by the enterotoxic activity of two large clostridial toxins, TcdA and TcdB, there are many additional factors that influence colonization, persistence and recurrence. However, the systematic investigation of non-toxin virulence factors lags considerably behind that of TcdA and TcdB. Accordingly, in this study we aimed at narrowing this gap by characterizing the PrsA2-homolog, a parvulin-type PPIase, of *C. difficile.*

PrsA of *B. subtilis* is important for the secretion of extracellular proteins (Kontinen et al., 1991; Jacobs et al., 1993; Yan and Wu, 2017; Ma et al., 2018). Homologs of PrsA in pathogenic bacteria very often contribute to pathogenicity by the secretion of extracellular virulence factors (Ünal and Steinert, 2014; Jiang et al., 2016; Wiemels et al., 2017; Lin et al., 2018). The most prominent example of these proteins is LmPrsA2 of *L. monocytogenes*, which has in addition to many other pathogens like *S. aureus* or *Streptococci* two homologs of this protein (Cahoon and Freitag, 2014). For our study, we identified all four putative parvulin-like PPIases of *C. difficile* and determined by sequence homology that CD630_35000 is the closest homolog to LmPrsA2. Additionally, CD630_35000 was confirmed as a lipoprotein in an experimental lipoproteomic approach (Charlton et al., 2015). Further analysis revealed that CdPrsA2, although closely related to the PrsA2 proteins of *B. subtilis* and *L. monocytogenes*, may have structural differences that might have functional consequences. The most interesting in this respect is certainly an additional 21 amino acid long stretch starting with K188 (Figure 1C). This stretch exhibits a certain similarity to a loop region in the human parvulin Pin1 that is involved in enzymatic activity and substrate discrimination (Zhou et al., 2000). In the light of these preliminary findings we decided to perform a systematic substitution analysis with conserved amino acids. Here, we demonstrated that the recombinant protein was indeed an active parvulin and that the most conserved amino acids within the PPIase domain, namely L241, F253 and Y273 are crucial for its enzymatic activity (Figure 2B). Substituting D232 with alanine resulted in increased PPIase activity suggesting that this residue also contributes to substrate selectivity. Interestingly, K188, which corresponds to K63 in human Pin1 but is not present in other PrsA2 proteins analyzed, also influences the activity of CdPrsA2. In Pin1, this positively charged residue together with R68 and R69 form a basic cluster which recognizes phospho-serine or phospho-threonine residues and facilitates its participation in mammalian signaling cascades (Zhou et al., 2000). With this substrate specificity Pin1 takes a special place among eukaryotic PPIases, whereas there are no reports for a selectivity toward phosphorylated amino acids in bacterial PPIases. Thus, we elucidated the substrate specificity of recombinant wild type CdPrsA2. Interestingly, we observed a clear preference of CdPrsA2 toward substrates containing positively charged amino acids preceding proline, which is similar to the substrate specificity of the second human parvulin Par14 (Uchida et al., 1999). Probably, K188 in CdPrsA2 does not contribute to substrate selectivity as K63 in Pin1. In contrast to Pin1, the 21 amino acid stretch of CdPrsA2 contains three aspartate residues and thus, might determine the preference for positively charged substrates together with D232. Also, it should be noted that CdPrsA2 might have a relatively broad substrate spectrum as the *cis/trans* isomerization of peptides with leucine, glutamine, methionine and phenylalanine preceding the proline was also accelerated at high rates.

In order to analyze the role of CdPrsA2 in *C. difficile* physiology, we generated a gene disruption mutant using the *ClosTron* technology. The mutant showed no major differences regarding growth in BHIS, sporulation on agar or toxin titres. Furthermore, the *prsA2::ClosTron* mutant and the wild type were comparably susceptible toward the cell wall targeting antibiotic vancomycin. In contrast, an increased susceptibility toward metronidazole, the second common first line antibiotic against CDI, was observed in the mutant (Figure 4B). This antibiotic that is administered against anaerobic pathogens exerts its activity mainly by causing DNA damage (Dhand and Snydman, 2009). In recent years, the resistance toward this considerably cheap antibiotic has constantly been rising among *C. difficile* isolates. However, the exact mechanisms conferring metronidazole resistance in *C. difficile* are not known. Suggested are increased activity of nitroreductases, iron uptake and DNA repair mechanisms (Peng et al., 2017). Among these, CdPrsA2 might influence the iron uptake by modulating the concentration or activity of membrane associated transporters.

Interestingly, in the absence of PrsA2 the resistance of *C. difficile* toward the secondary bile acids LCA and DCA significantly increased. The antibacterial effect of bile acids is a well-known phenomenon, as the probability and severity of acute CDI clearly correlate with the bile acid status of the host (Sung et al., 1993; Buffie et al., 2015). Nevertheless, the exact mode of action for the antibiotic activity of bile acids is still speculative. Considering their mainly hydrophobic nature, the disturbance of bacterial membranes is the most likely mode of action followed by interfering with the stability of cellular macromolecules like DNA, RNA or proteins. Most common bile tolerance or resistance mechanisms in bacteria include the modification of the outer membrane in Gram-negatives as well as the upregulation of transporters, efflux pumps or bile acid modifying enzymes in Gram-negative and Gram-positives (Begley et al., 2005). Many bacteria of the commensal microbiome, like *C. scindens*, are known to convert primary bile acids of the host to secondary bile acids that exert antibacterial activity on vegetative *C. difficile* and inhibit spore germination which contributes to the naïve colonization barrier in healthy individuals (Sorg and Sonenshein, 2009; Studer et al., 2016; Weingarden et al., 2016). In addition, especially the primary bile acid TCA and the secondary bile acid DCA induce germination of the spores, which is the key event leading to acute disease (Sorg and Sonenshein, 2008). How *C. difficile* might directly cope with bile acid stress is currently not known. Only recently a bile acid modifying 7α-hydroxysteroid dehydrogenase was biochemically characterized, while its contribution to bile acid dependent physiology of the bacterium remains unsolved (Bakonyi and Hummel, 2017). Considering the increase in resistance in the CdPrsA2-deficient mutant and a probable role of the protein in the composition of the outer layer of the bacterium, it is very likely that the interaction of LCA or DCA with the bacteria is hampered either by the reduced presence of a yet unknown receptor or a membrane modifying enzyme.

Most interestingly, PrsA2 deficiency resulted in a substantial loss in germination in response to the bile acids TCA and DCA (Figure 5). The germination is the initial step in the transformation to toxin producing vegetative cells and as such the development of acute disease. Germination in *C. difficile* differs from other clostridia and the model organism *B. subtilis* in regard to the regulatory cascades that are activated upon the detection of bile acids and amino acids as germination signals. Its main actors are the bile acid receptor CspC and the downstream proteases CspA, CspB, and SleC (Burns et al., 2010; Francis et al., 2013; Paredes-Sabja et al., 2014). How PrsA2 influences the germination of *C. difficile* still needs to be analyzed in detail, but the reduced response toward bile acids could also explain the reduced capability of the mutant to colonize mice. In orally infected mice the number of vegetative cells 24 h p.i. were significantly reduced in the cecum and colon. The same was true for the number of spores in the colon and feces, whereas interestingly the spore numbers in the cecum were comparable. This might indicate that the wild type due to a normal response toward bile acids can transform much earlier and more efficiently into vegetative cells and start the colonization. This early onset of infection most probably leads also to higher spore burden in the distal part of the gastrointestinal tract and finally in the feces. CdPrsA2, like all the other PPIases of *C. difficile*, has consistently been found in proteomic studies including the spore (Lawley et al., 2009; Charlton et al., 2015). However, CdPrsA2 was shown to be the only PPIase that was significantly upregulated throughout the first 30 h of infection in mice indicating that it actively participates in the infection process (Fletcher et al., 2018).

## Conclusion

We suggest that the extracellular PPIase PrsA2 of *C. difficile* is enzymatically active with a preference for substrates containing positively charged amino acids. Furthermore, it acts as a virulence modulator by influencing important processes including germination and bile acid resistance that all together contribute to the colonization efficiency of the bacteria. By this, it is part of the virulence repertoire of *C. difficile* whose pathogenic potential reaches beyond the toxic activity of TcdA and TcdB.

## Author Contributions

CÜ, CS-F, NS, MB, TS, DJ, and MS conceived the experiments. CÜ, CS-F, NS, MB, and CP conducted the experiments. CÜ, NS, and CS-F analyzed the data. CÜ and MS drafted and finalized the manuscript. All authors reviewed and approved the final manuscript.

## Conflict of Interest Statement

The authors declare that the research was conducted in the absence of any commercial or financial relationships that could be construed as a potential conflict of interest.

## Footnotes

1 http://www.clostron.com/
